# Supplementary material for: Production of leishmanin skin test antigen from Leishmania donovani for future reintroduction in the field
Source: Nat Commun. 2023 Nov 2;14:7028. doi: 10.1038/s41467-023-42732-2 (PMC10622560; doi:10.1038/s41467-023-42732-2)
Supplement: Supplementary file 1 — Supplementary Information [file 41467_2023_42732_MOESM1_ESM.pdf]

Production of Leishmanin Skin Test antigen from *Leishmania donovani* for future reintroduction in the field

SUPPLEMENTARY INFORMATION

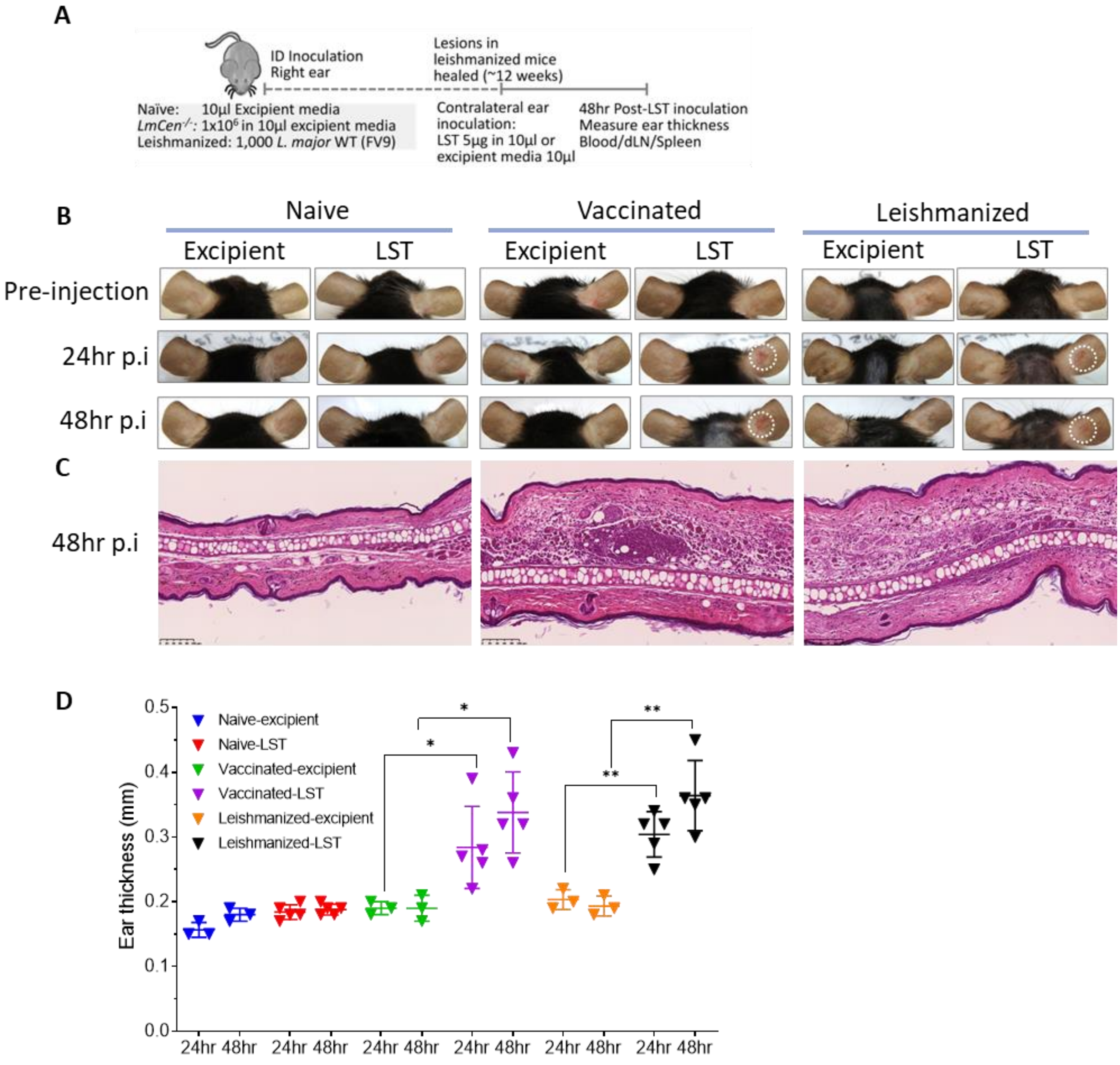

Supplementary Figure 1: Reproducibility testing *of L. donovani 1S2D* soluble antigen as a surrogate of immunogenicity. Healed wild-type *L. major FV9* infected (leishmanized) or

attenuated attenuated *LmCen*$^{-/-}$ immunized C57BL/6 mice were contralaterally injected with 5 µg of *L. donovani 1S2D* soluble antigen or excipient. The Leishmanin skin test (LST) response was evaluated at 24 and 48hr post-inoculation (pi). **A)** Illustration of the experimental design. **B)** Photographs of mice ears taken before, 24 and 48 hr post-injection of soluble antigens. **C)** Representative ears stained with H&E (×20) at 48hr post-injection of soluble antigens. Scale bars 20 µm. **D)** The corresponding ear thickness. Unpaired two tailed Student's *t* test was used to calculate statistical significance between 24hr vaccinated-excipient and vaccinated-LST groups (*p*=0.0486), 48hr vaccinated-excipient and vaccinated-LST groups (*p*=0.0083), 24hr leishmanized-excipient and leishmanized-LST groups (*p*=0.0037), 48hr leishmanized-excipient and leishmanized-LST groups (*p*=0.002). Results are shown as mean ± SD, and data is representative of two independent experiments with 3-5 mice per group. *, P ≤ 0.05; **, P ≤ 0.01; ***, P ≤ 0.001; and ****, P ≤ 0.0001 comparing groups. Source data are provided as a Source Data file.

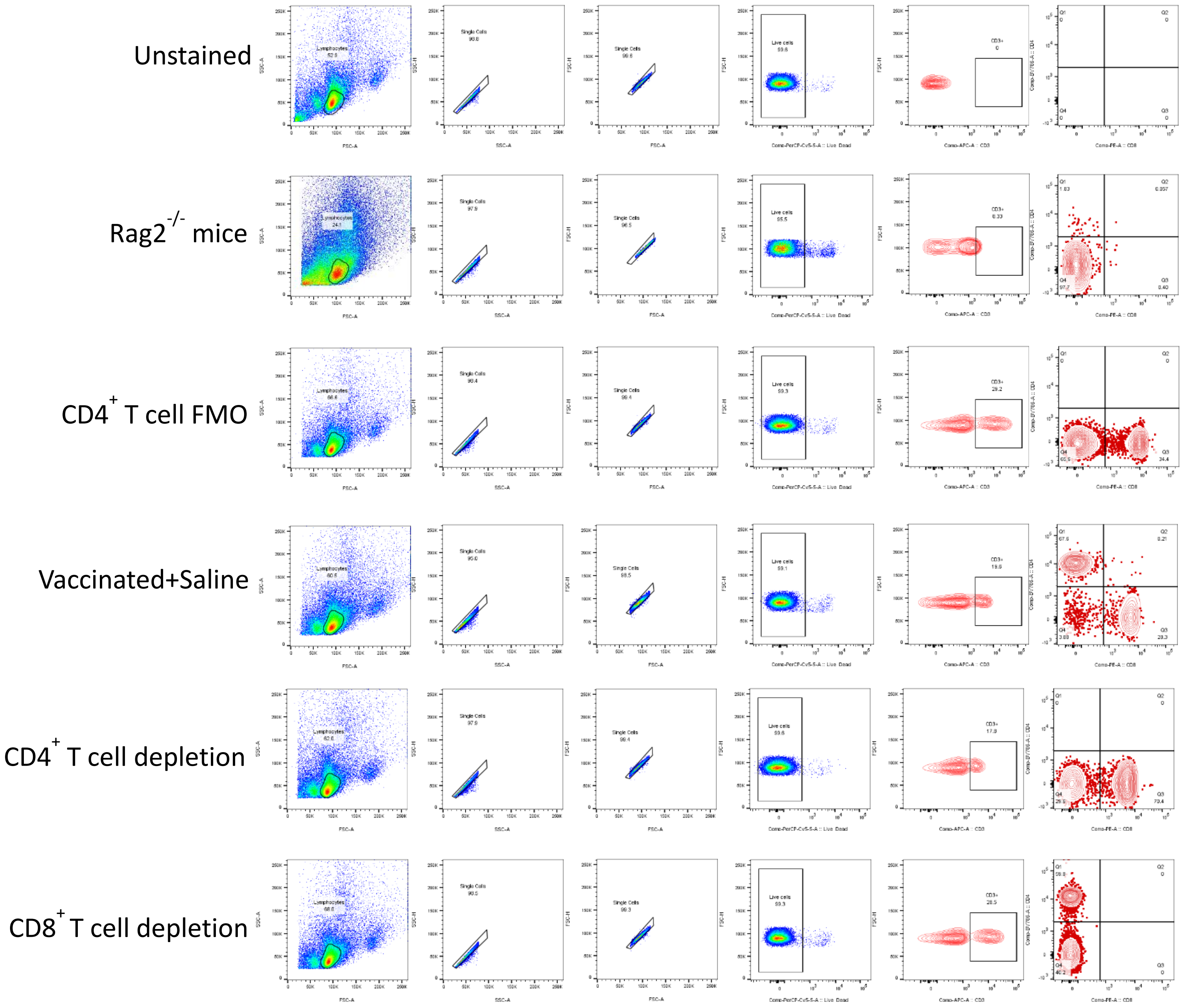

Supplementary Figure 2: $CD4^+$ or $CD8^+$ T cells were depleted in the wild-type *L. major FV9* infected (leishmanized) or attenuated *LmCen*$^{-/-}$ immunized C57BL/6 mice with a single intraperitoneal injection of 500 µg of anti-CD4 mAb (clone GK1.5) or 300 µg of anti-CD8 mAb (clone YTS169.4), respectively. The cells depletion was confirmed in some sampled mice using flow cytometry. Representative flow cytometric dot plots showing the gating strategy. FMO, Fluorescence Minus One.

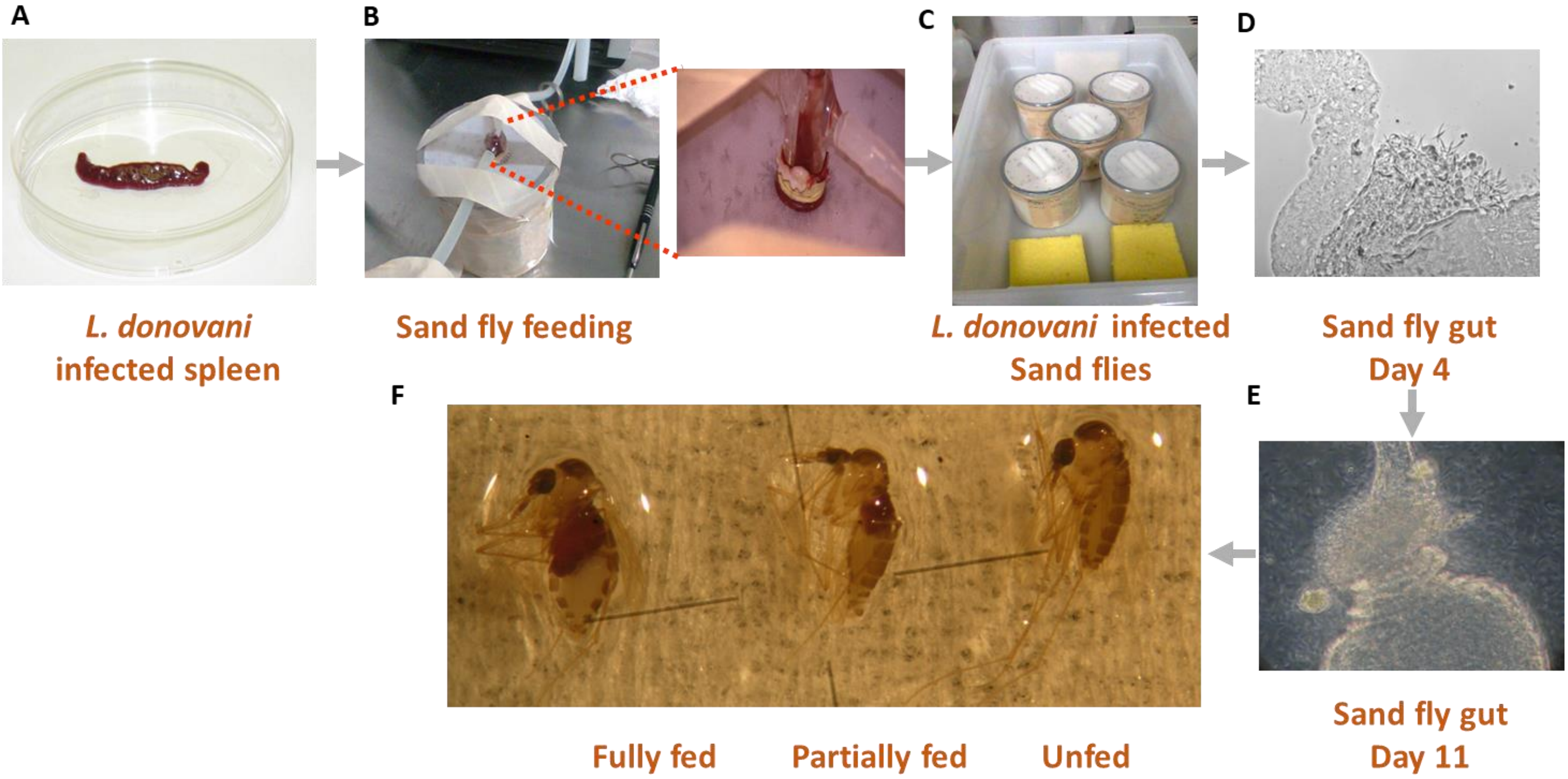

Supplementary Figure 3: Schematic of sand fly mediated infections of hamsters with *L. donovani* Ld-Ind

A) Spleen of a sick and moribund hamster infected with *L. donovani* Ld-Ind parasites was used as a source of parasites **B)** Sand files were fed through chicken membrane, a closeup of the ongoing feeding is shown **C)** Fully blood fed sand flies were isolated and were kept in 26^{0}C incubator **D)** At the indicated time points, a subset of sand flies was dissected and parasite growth in the midguts was monitored **E)** At day 11 post-feeding appearance of metacyclics at the anterior part of the gut indicated a mature infection and sand flies from such successful feeding were used to expose naïve hamsters **F)** Exposed sand flies were checked under microscope to count numbers of fully fed sand flies

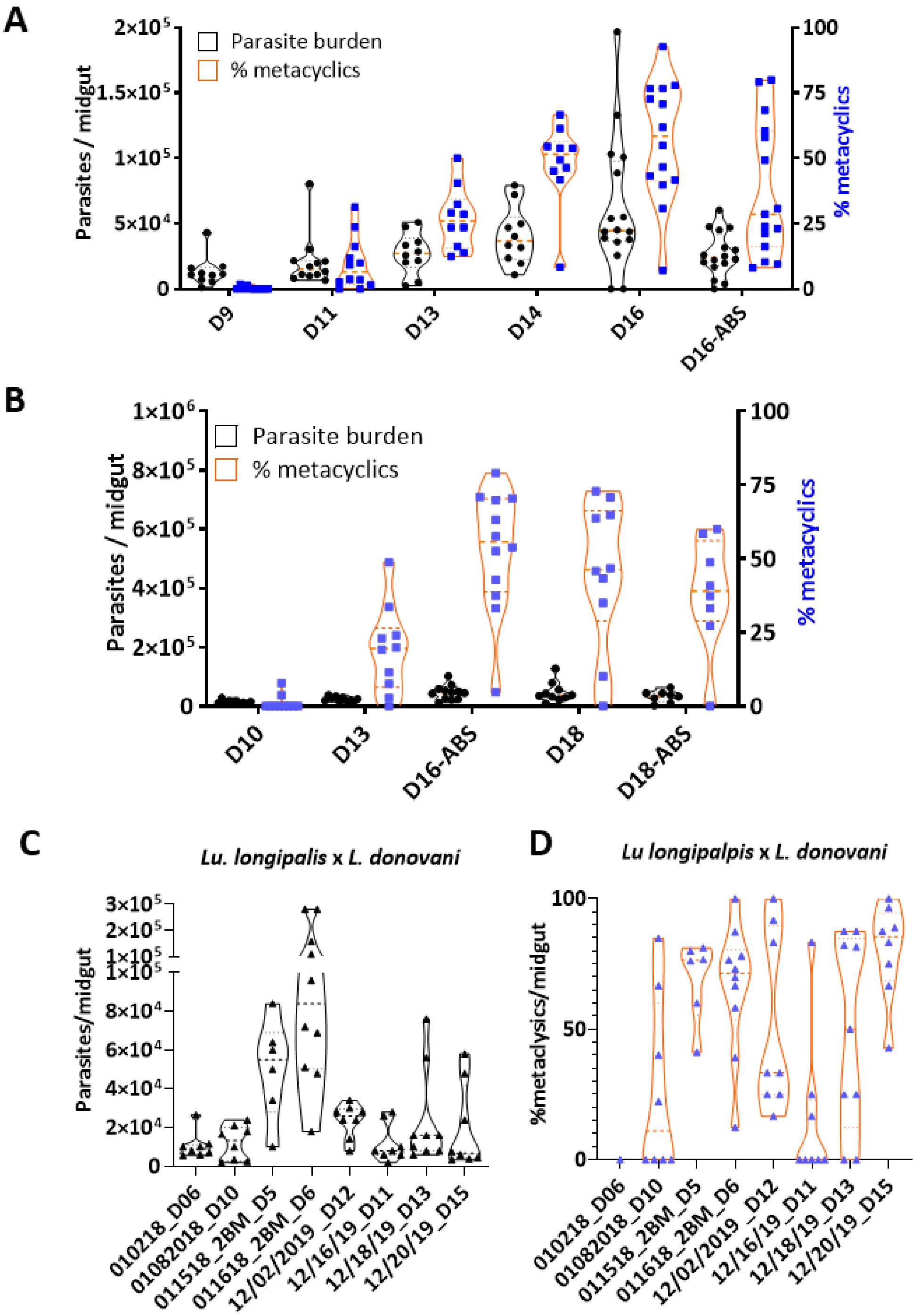

Supplementary Figure 4: Reproducible cycling of *L. donovani* Ld-Ind through sand flies

At different days of post artificial feeding through chicken membrane, sand flies were dissected, whole gut were homogenized in 50µl of PBS containing 0.1% formalin and the parasite numbers

were counted. A, B, C and D panels depict three independent experiments. Source data are provided as a Source Data file.

A

Lot #	Cell density $x10^6$/ml	Protein concentration after homogenization mg/ml
1	1100	2.68
2	750	1.32
3	1000	2.00

B

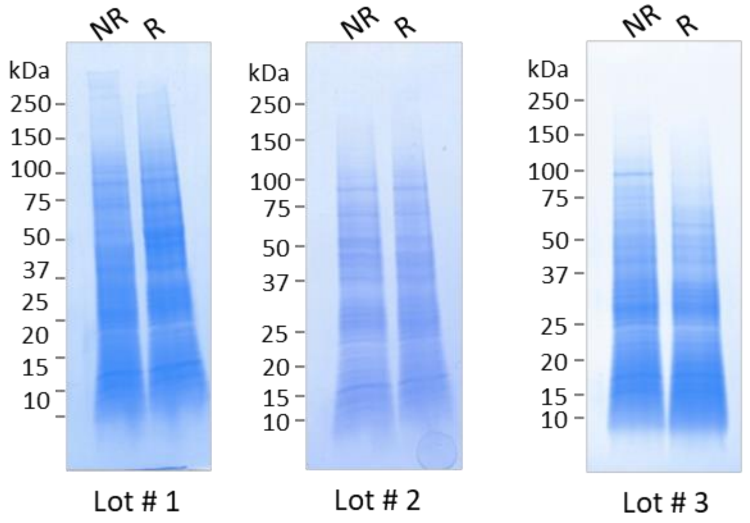

Supplementary Figure 5: Reproducibility of parasite growth and antigen preparation in three different manufactured lots

A) Parasite cultures in three additional manufactured lots showed consistent growth patterns in the bioreactor and yielded ≥750x10^6 and a protein concentration ≥1.32 mg/ml. B) SDS-PAGE analysis showed a consistent banding pattern of GLP-LST antigens from three additional manufactured lots. NR, non-reducing. R, reducing.
