# Supplementary material for: Production of leishmanin skin test antigen from Leishmania donovani for future reintroduction in the field
Source: Nat Commun. 2023 Nov 2;14:7028. doi: 10.1038/s41467-023-42732-2 (PMC10622560; doi:10.1038/s41467-023-42732-2)
Supplement: Supplementary file 3 — Reporting Summary [file 41467_2023_42732_MOESM3_ESM.pdf]

nature portfolio

Corresponding author(s): Greg Matlashewski

Last updated by author(s): Oct 13, 2023

Reporting Summary

Nature Portfolio wishes to improve the reproducibility of the work that we publish. This form provides structure for consistency and transparency in reporting. For further information on Nature Portfolio policies, see our Editorial Policies and the Editorial Policy Checklist.

Statistics

For all statistical analyses, confirm that the following items are present in the figure legend, table legend, main text, or Methods section.

n/a	Confirmed	
☐	☒	The exact sample size (n) for each experimental group/condition, given as a discrete number and unit of measurement
☐	☒	A statement on whether measurements were taken from distinct samples or whether the same sample was measured repeatedly
☐	☒	The statistical test(s) used AND whether they are one- or two-sided<br>*Only common tests should be described solely by name; describe more complex techniques in the Methods section.*
☒	☐	A description of all covariates tested
☒	☐	A description of any assumptions or corrections, such as tests of normality and adjustment for multiple comparisons
☐	☒	A full description of the statistical parameters including central tendency (e.g. means) or other basic estimates (e.g. regression coefficient) AND variation (e.g. standard deviation) or associated estimates of uncertainty (e.g. confidence intervals)
☐	☒	For null hypothesis testing, the test statistic (e.g. F, t, r) with confidence intervals, effect sizes, degrees of freedom and P value noted<br>*Give P values as exact values whenever suitable.*
☒	☐	For Bayesian analysis, information on the choice of priors and Markov chain Monte Carlo settings
☒	☐	For hierarchical and complex designs, identification of the appropriate level for tests and full reporting of outcomes
☒	☐	Estimates of effect sizes (e.g. Cohen's d, Pearson's r), indicating how they were calculated

Our web collection on statistics for biologists contains articles on many of the points above.

Software and code

Policy information about availability of computer code

Data collection	No software was used for data collection
Data analysis	Burrows-Wheeler Aligner (version 0.7.1734), file formats transformed using samtools (version 1.1035), and variant calling was done with VarScan2 (version 2.4.336) to generate VCF files. Circcos v0.69-8 , bcftools 1.16

For manuscripts utilizing custom algorithms or software that are central to the research but not yet described in published literature, software must be made available to editors and reviewers. We strongly encourage code deposition in a community repository (e.g. GitHub). See the Nature Portfolio guidelines for submitting code & software for further information.

Data

Policy information about availability of data

All manuscripts must include a data availability statement. This statement should provide the following information, where applicable:

- Accession codes, unique identifiers, or web links for publicly available datasets
- A description of any restrictions on data availability
- For clinical datasets or third party data, please ensure that the statement adheres to our policy

The sequencing data for the Ld-Ind-clone1,3 and 4 have been deposited at Genbank's sequencing read archive (SRA) as BioSamples SAMN31408468 [https://www.ncbi.nlm.nih.gov/biosample/SAMN31408468], SAMN31408469 [https://www.ncbi.nlm.nih.gov/biosample/SAMN31408469] and SAMN31408470 [https://www.ncbi.nlm.nih.gov/biosample/SAMN31408470] respectively, under the PRJNA893015 [https://www.ncbi.nlm.nih.gov/bioproject/PRJNA893015] BioProject. The

LdCL reference genome is available at TriTrypDB [https://tritrypdb.org/tritrypdb/app/record/dataset/TMPTX_ldonCL-SL]. Flow cytometry data has been deposited at FlowRepository under accession FR-FCM-Z6UG [http://flowrepository.org/id/FR-FCM-Z6UG]. Source data are provided with this paper.

Research involving human participants, their data, or biological material

Policy information about studies with human participants or human data. See also policy information about sex, gender (identity/presentation), and sexual orientation and race, ethnicity and racism.

Reporting on sex and gender	N/A
Reporting on race, ethnicity, or other socially relevant groupings	N/A
Population characteristics	N/A
Recruitment	N/A
Ethics oversight	N/A

Note that full information on the approval of the study protocol must also be provided in the manuscript.

Field-specific reporting

Please select the one below that is the best fit for your research. If you are not sure, read the appropriate sections before making your selection.

☒ Life sciences ☐ Behavioural & social sciences ☐ Ecological, evolutionary & environmental sciences

For a reference copy of the document with all sections, see nature.com/documents/nr-reporting-summary-flat.pdf

Life sciences study design

All studies must disclose on these points even when the disclosure is negative.

Sample size	Sample size calculations not relevant for this study. Samples size was determined based on pilot experiments that determined the dose of the antigens and duration of the DTH response in mouse models of leishmanization. Based on these results we included sufficient number of animals in the groups. Moreover the experiments were performed in both FDA laboratory in the USA and Nagasaki laboratory in Japan with identical results.
Data exclusions	There was no data exclusions in this manuscript
Replication	All experiments were replicated 3-4 times using different animal models and methods of challenge and we confirm that attempts to replicate the findings were successful
Randomization	Randomization was not relevant in this study as the results are qualitative and do not measure efficacy
Blinding	Blinding was not needed or used in this study as the phenotype change is beyond what can be affected by human bias

Reporting for specific materials, systems and methods

We require information from authors about some types of materials, experimental systems and methods used in many studies. Here, indicate whether each material, system or method listed is relevant to your study. If you are not sure if a list item applies to your research, read the appropriate section before selecting a response.

Materials & experimental systems

n/a	Involved in the study	
☐	☒	Antibodies
☐	☒	Eukaryotic cell lines
☒	☐	Palaeontology and archaeology
☐	☒	Animals and other organisms
☒	☐	Clinical data
☒	☐	Dual use research of concern
☒	☐	Plants

Methods

n/a	Involved in the study	
☒	☐	ChIP-seq
☐	☒	Flow cytometry
☒	☐	MRI-based neuroimaging

Antibodies

Antibodies used	Product Cat NO manufacturer Dilution Factor<br>TruStain FcX™ (anti-mouse CD16/32) Antibody 101320 Biolegend 1/100<br>7-AAD Viability Staining Solution 420404 Biolegend 5 µL/ Sample<br>APC-Cy7 Anti-mouse CD45 Antibodies; Clone QA17A26 157618 Biolegend 1/300<br>APC anti-mouse CD3ε Antibody; Clone 145-2C11 100312 Biolegend 1/200<br>BV786 Rat Anti-mouse CD4, clone GK1.5 563331 Biolegend 1/200<br>PE anti-mouse CD80 Antibody; clone 16-10A1 104708 Biolegend 1/200<br>Cell Staining Buffer 420201 Biolegend<br>InVivoMAb anti-mouse CD4 (Clone GK1.5) BE0003-1 BioXcell 500 µg/100 µL/ mouse<br>InVivoMAb anti-mouse CD8α (Clone YTS169.4) BE0117 BioXcell 300 µg/100 µL/ mouse
Validation	The antibodies have been titrated and described in previous publications from our laboratory (HL Nakhasi).<br>Front Immunol. 2018 Jun 4;9:1176. doi: 10.3389/fimmu.2018.01176. eCollection 2018. PMID: 29915577,<br>Front Immunol. 2017 Dec 12;8:1788. doi: 10.3389/fimmu.2017.01788. eCollection 2017. PMID:29312315

Eukaryotic cell lines

Policy information about cell lines and Sex and Gender in Research

Cell line source(s)	L. major Friedlin (FV9), L. donovani Ld-Ind,
Authentication	L. major Friedlin (FV9) confirmed to be L. major through genome sequencing previously, L. donovani Ld-Ind sequenced within
Mycoplasma contamination	not tested
Commonly misidentified lines<br>(See ICLAC register)	none

Animals and other research organisms

Policy information about studies involving animals; ARRIVE guidelines recommended for reporting animal research, and Sex and Gender in Research

Laboratory animals	Female 6- to 8-wk-old C57BL/6 mice, and female 6-8 week Rag2-/- mice, Six to eight-week-old female outbred Syrian golden hamsters (Mesocricetus auratus), Colony-bred 2- to 4-day-old Lutzomyia longipalpis females
Wild animals	No wild animals were used in this study
Reporting on sex	For comparison with previous animal experimental studies, only female mice and hamsters were used, moreover no studies have shown human leishmaniasis is affected by sex. Only female sandflies can transmit Leishmania, therefore only female sandflies were used in this study.
Field-collected samples	the study did not involve field collected samples
Ethics oversight	the Institutional Animal Care and Use Committee at the Center for Biologics Evaluation and Research, US FDA (ASP 1995#26, mouse protocol ASP-1999-23-hamster protocol), NIAID Animal Care and Use Committee under animal protocol LMVR4E, Institutional Animal Research Committee of Nagasaki University (No.1606211317 and 1505181227), the Nagasaki University Recombinant DNA Experiments Safety Committee (No. 1403041262 and 1407221278)

Note that full information on the approval of the study protocol must also be provided in the manuscript.

Plants

Seed stocks	N/A
Novel plant genotypes	N/A
Authentication	N/A

Flow Cytometry

Plots

Confirm that:

☒ The axis labels state the marker and fluorochrome used (e.g. CD4-FITC).

☒ The axis scales are clearly visible. Include numbers along axes only for bottom left plot of group (a 'group' is an analysis of identical markers).

☒ All plots are contour plots with outliers or pseudocolor plots.

☒ A numerical value for number of cells or percentage (with statistics) is provided.

Methodology

Sample preparation	As described in Materials and methods and sections there of.
Instrument	BD LSR Fortessa, BD FACS Aria-Fusion
Software	As described in Materials and methods and sections there of.
Cell population abundance	As described in Materials and methods and sections there of.
Gating strategy	Gating strategy for the flow cytometric analysis is described in supplementary figure S2

☒ Tick this box to confirm that a figure exemplifying the gating strategy is provided in the Supplementary Information.
